# Supplementary material for: A high-resolution mRNA expression time course of embryonic development in zebrafish
Source: eLife. 2017 Nov 16;6:e30860. doi: 10.7554/eLife.30860 (PMC5690287; doi:10.7554/eLife.30860)
Supplement: Supplementary file 6. [file elife-30860-supp6.zip › biolayout-clusters-files/Cluster167-genes.html]

Cluster167


# Cluster167: Genes

| Ensembl ID | Gene Name | Chr | Start | End | Biotype |
| --- | --- | --- | --- | --- | --- |
| ENSDARG00000098915 | CR318603.3 | 7 | 23894242 | 23901123 | protein\_coding |
| ENSDARG00000036420 | CR376787.1 | 7 | 20141283 | 20170713 | protein\_coding |
| ENSDARG00000075492 | ccdc160 | 14 | 31133552 | 31135077 | protein\_coding |
| ENSDARG00000038072 | ptx4 | 3 | 27511236 | 27532605 | protein\_coding |
| ENSDARG00000097580 | si:dkey-182m1.2 | 15 | 21586071 | 21604544 | processed\_transcript |
| ENSDARG00000097195 | si:dkey-202b22.7 | 4 | 18525481 | 18529624 | lincRNA |
| ENSDARG00000091950 | si:dkeyp-4c7.3 | 13 | 38100267 | 38152091 | protein\_coding |
| ENSDARG00000052244 | zgc:158564 | 7 | 39393038 | 39404655 | protein\_coding |
